# Combined “Test and Treat” Campaigns for Human Immunodeficiency Virus, Hepatitis B, and Hepatitis C: A Systematic Review to Provide Evidence to Support World Health Organization Treatment Guidelines

**DOI:** 10.1093/ofid/ofad666

**Published:** 2024-01-11

**Authors:** Natasha Beard, Andrew Hill

**Affiliations:** School of Medicine, School of Public Health, Imperial College London, London, United Kingdom; Department of Pharmacology and Therapeutics, University of Liverpool, Liverpool, United Kingdom

**Keywords:** blood-borne virus, hepatitis B, hepatitis C, HIV, triple testing

## Abstract

**Background:**

Worldwide, more than 39 million individuals are living with human immunodeficiency virus (HIV), 296 million with chronic hepatitis B (HBV), and 58 million with chronic hepatitis C (HCV). Despite successful treatments for these blood-borne viruses (BBVs), >1.7 million people die per annum. To combat this, the World Health Organization recommended implementing triple testing for HIV, HBV, and HCV. This systematic review aims to provide evidence for this policy, by identifying the prevalence of these BBVs and discussing the costs of available triple tests.

**Methods:**

Medline, Embase, and Global Health were searched to identify articles published between 1 January and 24 February 2023. Included studies reported the prevalence of HIV (anti-HIV 1/2 antibodies), HBV (hepatitis B surface antigen) and HCV (anti-HCV antibodies). Results were stratified into risk groups: blood donors, general population, healthcare attendees, individuals experiencing homelessness, men who have sex with men, people who use drugs, pregnant people, prisoners, and refugees and immigrants.

**Results:**

One hundred seventy-five studies sampling >14 million individuals were included. The mean prevalence of HIV, HBV, and HCV was 0.22% (standard deviation [SD], 7.71%), 1.09% (SD, 5.80%) and 0.65% (SD, 14.64%) respectively. The mean number of individuals testing positive for at least 1 BBV was 1.90% (SD, 16.82%). Therefore, under triple testing, for every individual diagnosed with HIV, another 5 would be diagnosed with HBV and 3 with HCV. Testing for all 3 viruses is available for US$2.48, marginally more expensive than the lowest-priced isolated HIV test ($1.00).

**Conclusions:**

This article highlights a potential avenue for healthcare improvement by implementing combination testing programs. Hopefully, this will help to achieve the Sustainable Development Goal of elimination of these BBV epidemics by 2030.

## Teaser

This systematic review investigated the prevalence of HIV, HBV, and HCV in 9 different population groups and found that if triple testing were to be implemented, for every case of HIV identified, another 5 cases of HBV and another 3 cases of HCV would be identified.

More than 84.2 million individuals have acquired human immunodeficiency virus (HIV) since the start of the epidemic, resulting in 40.4 million deaths [1]. The World Health Organization's (WHO) recommended first-line treatment for HIV—tenofovir/emtricitabine plus dolutegravir, which prolongs survival and prevents further viral transmission—is now available for less than US$50 per person per year, a cost-effective price for many countries [2, 3].

Chronic hepatitis B (HBV) affects 296 million individuals and is responsible for more than 40% of cirrhosis and 60% of hepatic cancer cases [4–6]. There have been several successful advances in controlling this epidemic, with a successful vaccine now available for only $0.49 per dose [7]. While chronic HBV can be treated for $28.80 per person/per year using generic tenofovir, currently only 2.2% of estimated infected individuals are receiving treatment [4, 8].

Hepatitis C (HCV) is a blood-borne RNA virus, and of those who acquire this virus, 70%–80% will develop chronic HCV, of which 15%–30% will develop cirrhosis [9]. Early diagnosis offers the greatest chance of cure, with a 12- to 24-week course of direct-acting antivirals (DAAs) achieving a cure in >95% of individuals [10]. While generic DAA sofosbuvir/daclatasvir is now available for $60 per person, these drugs were previously protected by expensive patents and could only be delivered in specialist-led hospitals, rendering them inaccessible to many [8–10].

Table 1 details the current circumstances for each epidemic. While all 3 viruses affect a similar number of individuals per annum, the combined number of deaths from HBV and HCV (1.11 million) is significantly greater than that of HIV (650 000), and it is estimated that by 2040 viral hepatitis will result in more deaths annually than the HIV, malaria, and tuberculosis epidemics combined [1, 4, 9, 11].

**Table 1.** Summary of the Current Human Immunodeficiency Virus, Hepatitis B Virus, and Hepatitis C Virus Epidemics, Including the Lowest-Priced Diagnosis and Treatment Options

Characteristic	HIV	HBV	HCV
Epidemic size	39 million	296 million	58 million
New infections per year	1.3 million	1.5 million	1.5 million
Deaths per year	650 000	820 000	290 000
Percentage diagnosed	85.0%	10.5%	21.0%
Percentage treated	75.0%	2.2%	13.0%
Treatment	Tenofovir/emtricitabine plus dolutegravir	Tenofovir	Sofosbuvir/daclatasvir
Cost of treatment (per person), US$	<$50.00/per year	$28.80/per year	$60.00
Diagnostic test	Anti-HIV 1/2 antibodies	Hepatitis B surface antigen	Anti-HCV antibodies
Cost of diagnostic test, US$	$1.00	$0.70	$0.78

Sources: [1, 3, 4, 5, 9, 11].

Abbreviations: HBV, hepatitis B virus; HCV, hepatitis C virus; HIV, human immunodeficiency virus.

Overlapping modes of transmission has also led to coinfection with these 3 blood-borne viruses (BBVs). It is estimated that 7.4% of people living with HIV are coinfected with HBV and 6.2% with HCV [4, 9]. Coinfection often accelerates the progression of these diseases, increasing the mortality rate beyond that of each virus as a monoinfection [12–14].

To combat these issues, as part of the Sustainable Development Goals (SDGs), the United Nations General Assembly pledged to end the HIV epidemic and combat hepatitis by 2030, with the WHO in 2023 also pledging to eliminate the HIV, HBV, and HCV epidemics by 2030 [15, 16]. These pledges are being tracked using the 95-95-95 target for HIV and 90-80 target for HBV and HCV [11, 16]. These targets are yet to be achieved, with most recent data showing progress of 86-89-93, 10.5-2.2, and 21-13 for HIV, HBV, and HCV respectively [1, 4, 9]. For each BBV, access to diagnostic tests is a significant barrier to achieving these targets; infected individuals cannot receive treatment if they are unaware of their disease status. The screening pathway for HIV has been successfully implemented worldwide, but those for hepatitis have fallen behind, with only 73% of the European Economic Area reporting testing guidance for HBV and/or HCV [17].

A potential solution to this disparity is triple testing, where an individual would be tested for all 3 BBVs, rather than just HIV. In 2022, the WHO released guidelines on HIV and hepatitis care in people who use drugs (PWUD), men who have sex with men (MSM), and prisoners, which recommended triple testing for these individuals [18]. However, there is limited literature comparing the efficacy and costs of offering triple test programs against stand-alone HIV programs. This systematic review aims to cover this gap, by determining the prevalence of HIV, HBV, and HCV monoinfections yielded through triple testing and discussing the costs of implementing such programs.

## METHODS

This is a systematic review of primary data, conducted following the Cochrane framework for systematic reviews and the Preferred Reporting Items for Systematic Reviews and Meta-Analyses (PRISMA) guidelines [19].

The inclusion and exclusion criteria for this article were synthesized using the PICOS (population, intervention, comparison, outcome, study design) framework, full details of which can be found in Supplementary Appendix 1.

Studies were included if they reported on the prevalence of HIV, HBV, and HCV. This may be reported from using either multiplex testing or 3 individual tests. Studies must have tested for these viruses using medically approved methods of identifying HIV, HBV, and HCV (ie, blood and serum samples). In this review, HIV positivity was defined as anti-HIV 1/2 antibody presence, HBV as hepatitis B surface antigen positivity, and HCV as anti-HCV antibody presence. While all studies had to use these diagnostic tests, the pharmaceutical provider of each test could differ between studies. These 3 markers were chosen as they are either pathognomic for chronicity or traditionally used for screening of their respective BBV [20–22]. Studies were excluded if participants self-reported their disease status, unless they were later verified by confirmatory medical tests.

While no constraints were placed on the population of interest in order to allow for later paper stratification, most of the included studies were conducted in adults (defined as aged ≥16 years). However, allowances were made for studies conducted in refugee cohorts, as children form 40% of this population [23].

To collate papers, 3 international databases (Medline, Embase, and Global Health) were searched for papers published between 1 January 2013 and 24 February 2023. This period was chosen to ensure our search would yield sufficient data, while also ensuring the conclusions drawn could be applied to the current global picture. Papers were only included if published in the English language. Supplementary Appendix 2 contains a detailed breakdown of the search strategy and key terms utilized.

All papers produced from the search strategy were uploaded onto COVIDence, a platform used to aid screening and data extraction. One reviewer screened each record. If the papers were deemed relevant, data extraction of the following characteristics, if available, was performed: year of publication, sample size, geographical location of study, mean/median age of population, sex, prevalence of HIV, HBV, and HCV, prevalence of dual and triple infection, and the total proportion of the population testing positive for 1 BBV. Where several papers reporting on the same country and category existed, the prevalence was determined by calculating the weighted mean across all studies.

Included papers were stratified according to their risk group. Studies were classified under pregnant women if their population were people whose pregnancy had been confirmed by a medical practitioner. Studies whose populations consisted of voluntary, paid, or familial blood donors were categorized into blood donors. While the WHO only recommends voluntary blood donations, in >25% of countries, paid and familial donors account for >50% of the donor pool, and therefore, papers including these donors were included to allow for global application of results [24]. Healthcare attendees consisted of studies whose participants had been tested while actively seeking healthcare, irrespective of their symptoms. Studies were categorized as MSM if >75% of the cohort self-identified as homosexual. Similarly, studies were classified as PWUD if >75% of the population self-reported drug use or had been admitted for medical treatment related to drug intake. Studies who were recruited from facilities commonly accessed by homeless persons (eg, hostels and shelters) were categorized under homeless individuals. Studies were classified as refugees and immigrants if the population self-identified as a refugee, immigrant or asylum seeker, or if the studies were conducted at recognized refugee registration centers. Likewise, studies were categorized as prisoners if the study was conducted at a recognized incarceration facility. The remaining studies were categorized into general population.

Included studies were assessed and rated according to their quality and risk of bias. The tool used was synthesized using a combination of the Institute of Health Economics Quality Appraisal Checklist for Case Series Studies and an assessment of bias tool created specifically for studies reporting on disease prevalence [25, 26]. Studies whose participants were recruited using either cluster or randomized sampling and reported sociodemographic characteristics were scored higher, whereas studies that did not report this data or sampled from small nonrandom and nonrepresentative populations scored lower. A full breakdown of the tool can be found in Supplementary Appendix 3.

All statistical tests and graphs were generated using Microsoft Excel Version 16.54. Where the mean of the data was required, the weighted mean was used to generate results. Choropleth maps were generated using DataWrapper.

The nature of this study meant that ethical approval was not required.

## RESULTS

A total of 1885 studies were identified and retrieved, of which 175 met the inclusion criteria. The process of paper identification and exclusion can be found in Supplementary Appendix 4. All of the extracted data, including sociodemographic characteristics and risk of bias score for the 175 studies, are available upon request.

Table 2 demonstrates the key findings from each population group. Across each BBV, similar trends can be seen. For HIV and HBV, the smallest burden can be seen in the general population (0.09% [standard deviation [SD], 4.28%] and 0.22% [SD, 3.46%], respectively) and the greatest burden among PWUD (20.47% [SD, 13.27%] and 9.87% [SD, 13.21%], respectively). This in keeping with total BBV prevalence, with the lowest prevalence among the general population (0.87% [SD, 8.96%]) and the highest among PWUD (60.56% [SD, 23.03%]). Interestingly, the greatest burden of coinfections can also been seen among PWUD at 24.83% (SD, 16.05%). HCV prevalence appears to be greatest again among PWUD (52.79% [SD, 20.62%]), with the lowest prevalence found in pregnant people (0.31% [SD, 1.71%]). Comparing the prevalence of HIV to each hepatitis, healthcare attendees showed the greatest disparity between HBV and HIV prevalence, with HBV being 40.37 times more prevalent than HIV. With regard to HIV and HCV, the biggest contrast can be seen among the general population, where HCV is 6.55 times more common than HIV. Across all population groups, HBV is almost 5 times and HCV almost 3 times more prevalent than HIV.

**Table 2.** Prevalence of Human Immunodeficiency Virus, Hepatitis B Virus, and Hepatitis C Virus by Population (Calculated Using Weighted Mean)

Category	No. of Studies	HIV Positive, % (SD)	HBV Positive, % (SD)	HCV Positive, % (SD)	1 BBV Positive, % (SD)	Coinfection, % (SD)	HBV:HIV	HCV:HIV
General population	11	0.09 (4.28)	0.22 (3.46)	0.60 (3.88)	0.87 (8.96)	0.05 (2.45)	2.44	6.55
Blood donors	43	0.33 (1.70)	1.13 (3.51)	0.53 (1.42)	1.95 (1.42)	0.54 (1.59)	3.45	1.61
Pregnant women	16	0.39 (3.17)	1.50 (4.02)	0.31 (1.71)	2.19 (6.14)	0.07 (1.30)	3.81	0.79
Healthcare attendees	34	0.18 (5.04)	7.21 (6.39)	1.14 (8.12)	8.52 (10.31)	0.21 (4.99)	40.37	6.38
Refugees/immigrants/asylum seekers	35	0.17 (1.76)	1.19 (4.04)	0.47 (2.27)	1.80 (5.61)	0.48 (0.88)	7.03	2.75
Prisoners	15	1.03 (2.03)	1.27 (3.37)	5.69 (6.82)	7.78 (6.76)	0.62 (0.48)	1.23	5.53
Homeless individuals	4	3.38 (2.31)	1.80 (0.70)	21.75 (8.68)	26.93 (10.86)	NA	0.53	6.44
MSM	4	18.28 (30.45)	1.26 (2.56)	1.42 (2.15)	20.08 (32.03)	3.47	0.07	0.08
PWUD	13	20.47 (13.27)	9.87 (13.21)	52.79 (20.62)	60.56 (23.03)	24.83 (16.05)	0.48	2.58
Total	175	0.22 (7.71)	1.09 (5.80)	0.65 (14.64)	1.90 (16.82)	0.12 (10.44)	4.93	2.96

Abbreviations: BBV, blood-borne virus; HBV, hepatitis B virus; HCV, hepatitis C virus; HIV, human immunodeficiency virus; MSM, men who have sex with men; NA, not applicable; PWUD, people who use drugs; SD, standard deviation.

**Figure 1.**
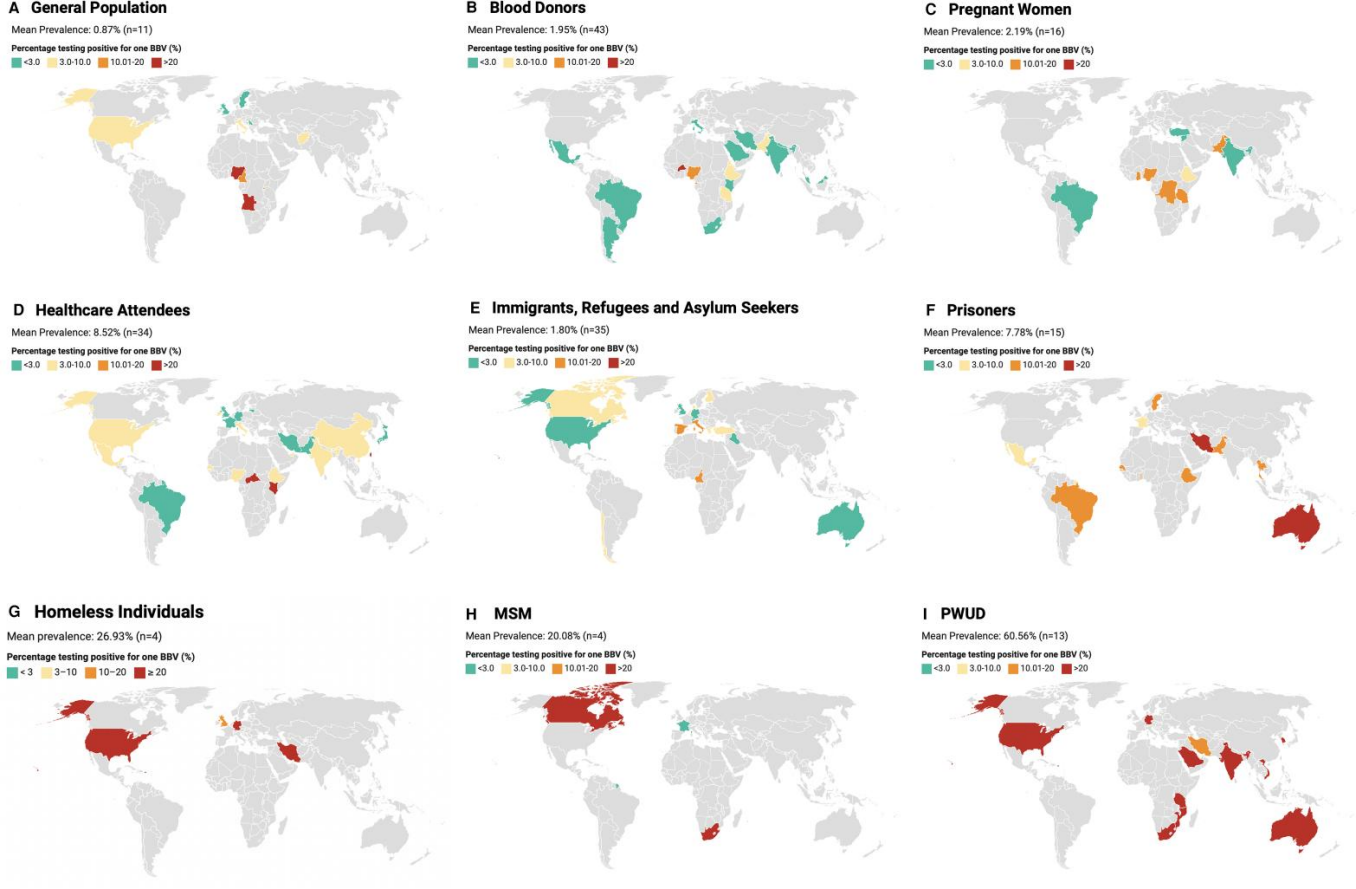
Mean prevalence and percentage testing positive for 1 blood-borne virus (BBV) among the general population (*A*); blood donors (*B*); pregnant women (*C*); healthcare attendees (*D*); immigrants, refugees, and asylum seekers (*E*); prisoners (*F*); homeless individuals (*G*); men who have sex with men (MSM; *H*), and people who use drugs (PWUD; *I*).

Figure 1 shows the geographical distribution of BBV prevalence across the 9 subpopulations, and highlights the disparities in BBV prevalence between countries. A prevalence of less than 3% is highlighted in green, as a previous article by Orkin et al reported a total BBV prevalence of 3.35% [27]. In Figure 1*A*–*D*, the majority of countries are green or yellow, reflecting less than 10% of the population testing positive for 1 BBV. However, there is significant heterogeneity between WHO regions in these populations. Countries within the Western Pacific Region, Region of the Americans, and European Region are all green (<3% prevalence), whereas countries within the African Region, South-East Asian Region, and Eastern Mediterranean Region contain a mixture of green (<3% prevalence), orange (10%–20%), and red (≥20%). For example, in Figure 1*A*, every country within African Region showed that at least 10% of the general population tested positive for 1 BBV.

Similarly, in Figure 1*E*, 1*F*, and 1*H*, representing immigrants, refugee and asylum seekers, prisoners, and MSM, respectively, there is a greater global heterogeneity in prevalence, ranging from <5% of said population testing positive for 1 BBV to >20% in some countries. Within these populations, there are countries within the Western Pacific Region, Region of the Americas, and European Region that are orange and red, representing at least 10% of the population testing positive for 1 BBV. Figures 1*G* and 1*I* only display red or orange, meaning at least 15% of homeless individuals and 10% of PWUD test positive for 1 BBV, in each country where data were provided.

**Figure 2.**
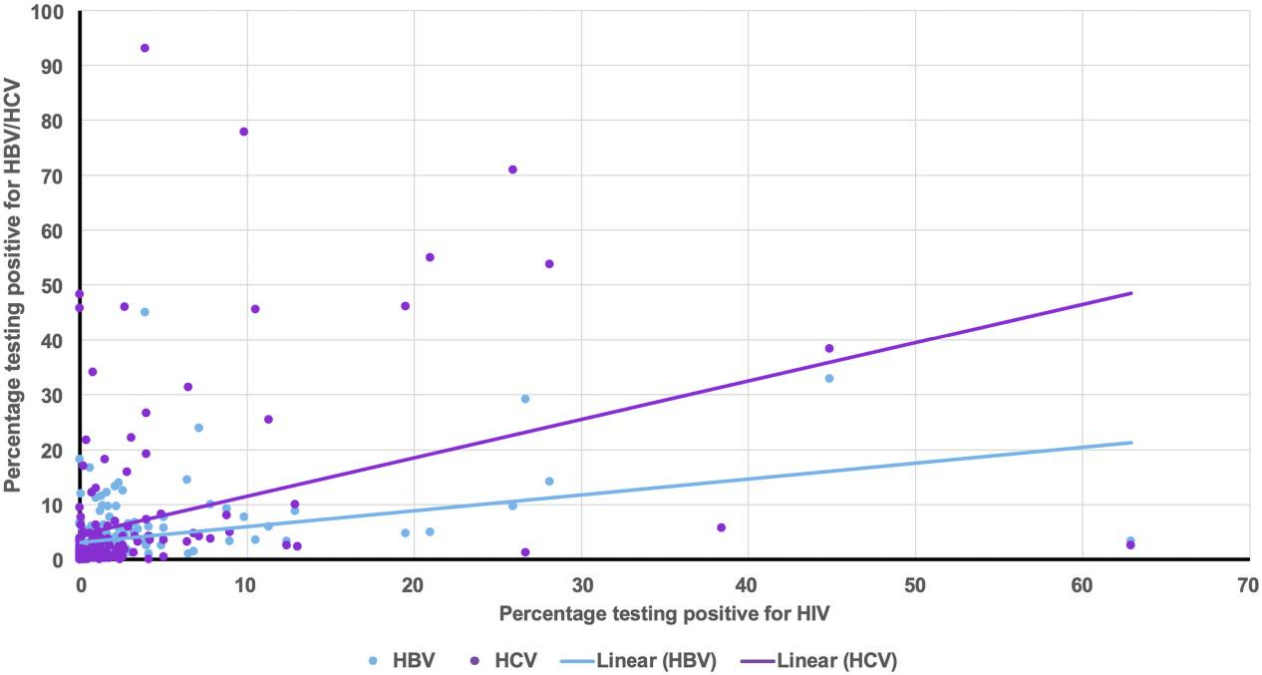
A scatter graph showing the relationship between human immunodeficiency virus (HIV)/hepatitis B virus (HBV) and HIV/hepatitis C virus (HCV) prevalence.

Figure 2 reflects the relationship between HIV/HBV and HIV/HCV prevalence across studies. It shows that as HIV prevalence increases, so does HBV and HCV prevalence across all vulnerable groups. However, HCV prevalence appears to increase at a more dramatic rate compared to HBV at the same HIV prevalence. While the vast majority of studies cluster around the <10% prevalence, there are several outliers, especially with regard to HIV/HCV prevalence.

**Figure 3.**
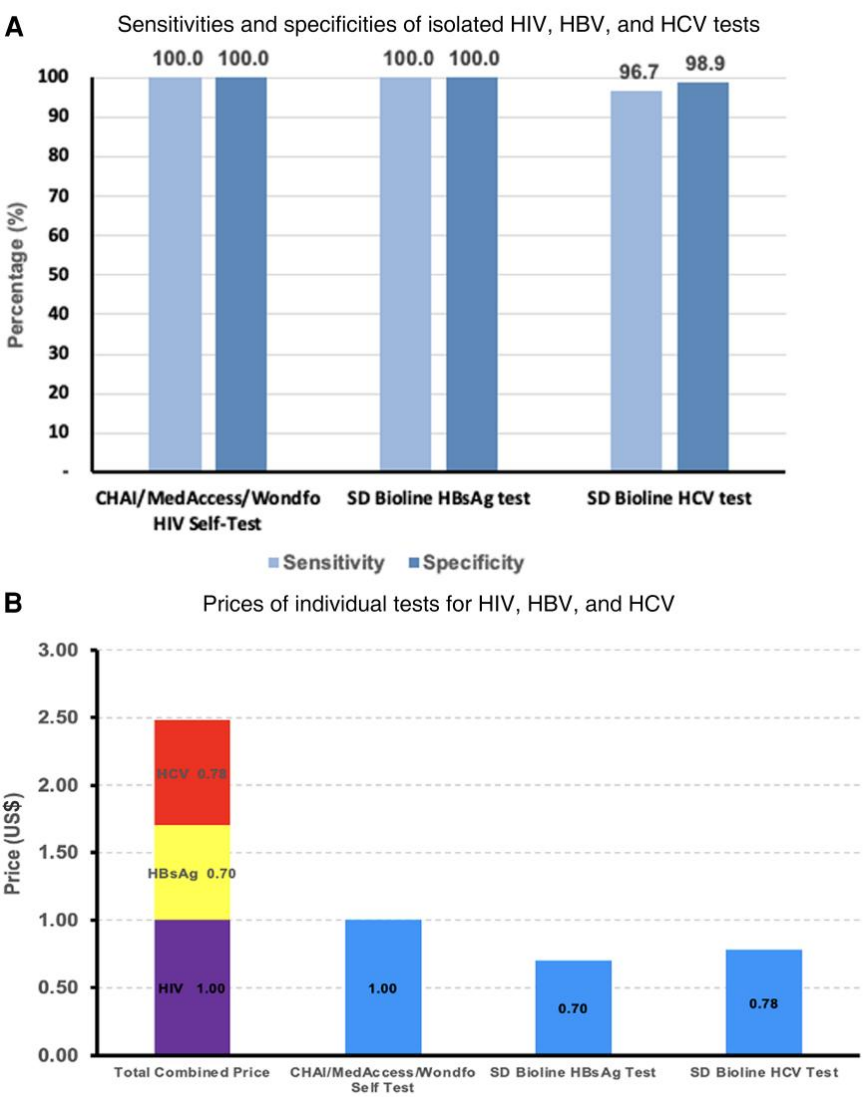
*A*, Sensitivities and specificities of the lowest-priced isolated human immunodeficiency virus (HIV), hepatitis B virus (HBV), and hepatitis C virus (HCV) tests. *B*, Comparison of individual tests for HIV, HBV, and HCV. These prices are all in US$. For HIV, the test used was the Clinton Health Access Initiative (CHAI)/MedAccess/Wondfo HIV Self-Test, for HBV the SD Bioline hepatitis B surface antigen (HBsAg) test, and for HCV the SD Bioline HCV Test [28–30]. These were the lowest-priced World Health Organization–approved tests for their respective blood-borne virus according to the literature.

Figure 3 reflects the costs and sensitivities/specificities of the lowest-priced HIV, HBV, and HCV tests available [28–30]. These tests are all WHO prequalified, and Figure 3 reflects each test's sensitivities and specificities. Using these prices, the total cost of testing for all 3 viruses is $2.48, a price that is marginally more expensive than the lowest-priced HIV test ($1.00) [28, 30].

## DISCUSSION

In this analysis of 175 studies including >14 million individuals, the combined prevalence of HBV (1.09%) and HCV (0.65%) is consistently greater than the prevalence of HIV (0.22%) in every category except MSM. This provides justification for the expansion of isolated HIV testing into triple BBV testing in not just the WHO's recommended risk groups, but for all vulnerable groups mentioned. These results also demonstrate that via multiplex testing, for every 1 HIV case identified, another 5 cases of HBV and 3 HCV cases would be diagnosed.

These findings are in concordance with the current literature. One systematic review reported HIV, HBV, and HCV prevalences of 1.00%, 12.07%, and 2.74%, respectively, while another found rates of 3.1%, 23.4%, and 19.6%, respectively [31, 32]. These papers, alongside this systematic review, demonstrate the significant burden of disease that hepatitis, especially HBV, poses in comparison to HIV.

Analyzing the geographical location of results reveals common themes. In groups that are referred to as “low risk” (blood donors, pregnant people), and the general population, the prevalence of BBV(s) is greatest among the African Region. However, in groups that are referred to as “high risk” (prisoners, PWUD), the prevalence is predominantly greatest in the Western Pacific Region. This in agreement with existing knowledge. In low-income countries, 89% of which are in the African Region, many individuals face absolute poverty, meaning they cannot afford to access diagnostic and treatment schemes for these BBVs, leaving the whole population vulnerable to disease [33]. However, in high-income countries, such as those in the Western Pacific Region, adequate medical infrastructure is available to the entire community, so diseases cluster into smaller groups, where social behaviors are predominantly responsible for disease acquisition.

This review has several strengths. It is believed to be the first systematic review to report on and compare the prevalence of HIV, HBV, and HCV yielded through triple testing within these 9 vulnerable populations. While there is heterogeneity in results between groups, this paper consistently found a greater HBV and HCV prevalence than that of HIV in all populations except MSM, reflecting sound methodology.

However, there are some limitations. Due to author constraints, only English-language studies were included. This results in potential positive discrimination toward English-speaking countries, which are typically high-income countries, decreasing the reliability of application of results on a global scale.

Furthermore, there was significant variation between countries’ representation. While this study encompassed 56 of 193 countries, some were represented in only one risk group, whereas others provided multiple estimates for the same category (eg, India provided 13 studies for blood donors). Additionally, sometimes only one country would provide data for their entire WHO region. This creates difficulty in accurately comparing BBV rates between regions and countries. However, as the scope of this paper was to compare HIV, HBV, and HCV prevalence within each category, as opposed to between them, this limitation can be utilized as an area for further research, to allow for direct comparison of BBV prevalence between categories.

In this article, HCV positivity was defined as the presence of anti-HCV antibodies, whereas the gold standard diagnostic test for chronic infection is a test for HCV RNA, after which treatment is traditionally commenced [9]. It is estimated that 70% of people who test positive for anti-HCV antibodies also test positive for HCV RNA [9]. Applying this estimate to the results in this article means that for every HIV case, another 2 cases of HCV would be identified, still providing justification for the WHO's recommendation. Additionally, a large proportion of people with HCV often face significant social barriers, which may limit the ability to measure HCV RNA due to loss to follow-up and failure of linkage to care. HCV RNA tests are also vastly more expensive than anti-HCV antibody tests ($8.50 vs $0.78, respectively), limiting accessibility [ 30, 34]. As a result, some nations, such as China, are no longer requiring HCV RNA testing to diagnose chronic infection, and further research will be required to determine the clinical and financial effectiveness of this scheme, to determine global feasibility [35].

While this review has demonstrated that under isolated HIV testing 8 cases of hepatitis would remain undiagnosed for every positive HIV case, it is important to not overlook the barriers concerning the practicalities of implementing global testing. To be effective, not only do these low-price and largely affordable tests need to be made accessible to all risk groups globally, but healthcare staff need to be adequately trained to deliver these tests, and universal access to appropriate and affordable treatment must be provided. These low cost HBV and HCV treatments are not universally accessible, with HBV and HCV treatments being priced around $7000 and $75 000, respectively, for a pharmaceutical company in the United States, thus potentially limiting access to those most vulnerable [36, 37]. Furthermore, these schemes need to be culturally appropriate to both the region and the vulnerable individuals they are serving. In many countries, religious and cultural beliefs often influence BBV stigmatization, creating difficulties in increasing the uptake and acceptance of testing, irrespective of test availability. It is only if these problems, among many others, are also addressed, that the 2030 targets will be achieved.

This review showed that testing for HIV, HBV, and HCV using 3 individual tests would only be marginally more expensive than isolated HIV testing. These tests in Figure 3 are WHO approved and low cost, providing an immediate option for triple test expansion. While combination HIV/HBV/HCV triple tests are now being developed, and are available for only $1, these tests are yet to be approved by the WHO [38]. Therefore, while further research is undertaken to obtain approval for these tests, individually testing for HIV, HBV, and HCV is a low-cost interim solution for the WHO's recommendation.

Although the scope of this review predominantly concerned diagnostic pathways, further research should be undertaken to assess the feasibility of expansion into preventive measures. For example, individuals who test negative could be offered the hepatitis B vaccination, which provides 98%–100% protection against HBV, or event-driven preexposure prophylaxis for HIV, which can be provided for <$12 per person per year [4, 39]. Further research is also required to understand if other infections could also be tested for. For example, syphilis infection, which has the same transmission route as the aforementioned BBVs, can be tested for alongside these viruses. Therefore, the only cost of introducing this infection into the program would be through the laboratory test, which can be provided for $2.70 [40].

## CONCLUSIONS

This article highlights the necessity of implementing triple testing for HIV, HBV, and HCV globally. With the number of people dying from hepatitis now exceeding that of HIV, the exclusionary use of isolated HIV tests only paves way for the HBV and HCV epidemics to continue to grow. It is only through extensive and affordable diagnosis that the WHO's targets and the SDGs will be achieved and for a further 1.7 million deaths to be prevented.

## Supplementary Material

ofad666_Supplementary_Data

## Supplementary Data

Supplementary materials are available at *Open Forum Infectious Diseases* online. Consisting of data provided by the authors to benefit the reader, the posted materials are not copyedited and are the sole responsibility of the authors, so questions or comments should be addressed to the corresponding author.

## Notes

***Acknowledgments.*** The authors acknowledge Dr Samuel Cross and Dr Toby Pepperrell for their help in sourcing financial information with regards to the tests.

***Patient consent.*** All studies included in this systematic review were approved by local ethics committees, and all patients gave informed consent.

***Financial support*.** This research is supported by the International Treatment Preparedness Coalition’s Make Medicines Affordable Campaign (grant number ITPC/MV/2021 to N. B. and A. H.).
