# Supplementary material for: Combined “Test and Treat” Campaigns for Human Immunodeficiency Virus, Hepatitis B, and Hepatitis C: A Systematic Review to Provide Evidence to Support World Health Organization Treatment Guidelines
Source: Open Forum Infect Dis. 2024 Jan 11;11(2):ofad666. doi: 10.1093/ofid/ofad666 (PMC10863549; doi:10.1093/ofid/ofad666)
Supplement: ofad666_Supplementary_Data [file ofad666_supplementary_data.docx]

***Appendices***

***Appendix 1: PICOS Inclusion and Exclusion Criteria***

|  | **Inclusion** | **Exclusion** |
| --- | --- | --- |
| **Population** | Studies with at least 50 participating individuals  General population or at-risk key populations | Paediatric populations (mean age of population under 16 years of age), except for studies conducted in refugee or asylum seeker populations  Populations smaller than 50 participants |
| **Intervention** | Studies testing for the following variables:   - HIV: anti-HIV 1/2 antibody - HBV: Hepatitis B surface antigen - HCV: anti-HCV antibody   These tests could be either:   - Blood (whole blood, serum, plasma) - Saliva   Both venepuncture and dried blood sample tests were included.  Tests could be performed either by a medical professional in a healthcare facility, or as self-tests at home | Self-reported disease status (unless later confirmed through medical tests)  Studies that defined disease status using different variables   - HIV: HIV NAT - HBV: anti-HBs, anti-HBc - HCV: HCV RNA |
| **Comparator** | N/A | N/A |
| **Outcome** | Prevalence of HIV, HBV and HCV | Prevalence of one or two of HIV, HBV or HCV  Studies only reporting incidence of HIV, HBV and HCV  Any studies not reporting the prevalence of HIV, HBV and HCV |
| **Studies** | Human studies  Epidemiological studies measuring prevalence  Abstracts from posters/oral presentations | Animal/cell/non-human studies  Other systematic reviews or meta-analyses  Simulation/modelling studies  Studies not reported in the English language  Studies published before 1^st^ January 2013 |

***Appendix 2: Search Strategy***

|  | **MeSH terms (MEDLINE, Embase, Global Health)** |
| --- | --- |
| 1 | HIV infections/diagnosis |
| 2 | Hepatitis B/diagnosis |
| 3 | Hepatitis C/diagnosis |
| 4 | Humans |
| 5 | 1 AND 2 |
| 6 | 1 AND 3 |
| 7 | 2 AND 3 |
| 8 | 1 AND 2 AND 3 |
| 9 | 5 OR 6 OR 7 OR 8 |
| 10 | 4 AND 9 |
| 11 | Screening (for MEDLINE, the MeSH term used was mass screening) |
| 12 | 10 AND 11 |

| Risk of Bias | Criteria for answers | Additional Information |
| --- | --- | --- |
| External Validity | | |
| 1.)Was the study’s target population a close representation of the national population in relation to relevant variables e.g. age, sex | 2 points= The study was a close representation of the national population  1 point=The study was not a close representation; participants were taken from villages/only a few provenances  0 points= No data was provided |  |
| 2.)Was the study’s sampling frame a true or close representation of the target population | 2 points=cluster sampling was used, or every eligible individual was sampled  1 point= sampling method was detailed, but was not either of the above  0 points=sampling method not mentioned |  |
| 3.)Was the likelihood of non-response bias minimal? | 1 point= Yes. The response rate was ≥75% OR analysis of relevant characteristics showed no significant difference between responders and non-responders  0 points= No. The response rate was <75% OR analysis showed significant difference between responders and non-responders OR no analysis was conducted |  |
| Internal Validity | | |
| 4.)Did the study use an acceptable case definition | 1 point=acceptable case definitions were provided for HIV, HBV and HCV  0 points= acceptable case definitions were provided for one or two of the above, or none of the above |  |
| 5.)Was the study instrument that measured the parameter of interest shown to have reliability and validity? | 2 points= standard and confirmatory assays were performed  1 point= standard tests were performed  0 points= paper did not specify what tests were performed | Definition of standard test:  -HIV: anti-HIV 1/2 antibodies  -HBV: Hepatitis B surface antigen  -HCV: anti-HCV antibodies |
| 6.)Were the numerator and denominator for the parameters of interest appropriate? | 1 point= Yes, they were appropriate and there were no errors in calculations  0 point= No, errors w0ere made in some of the calculations |  |
| 7.)Were the conclusions of the study supported by the results | 1 point= Yes  0 points= No |  |
| 8.)Were there any competing interests declared? | 1 point= No  0 points= Yes |  |

***Appendix 3: Risk of Bias Tool Used***


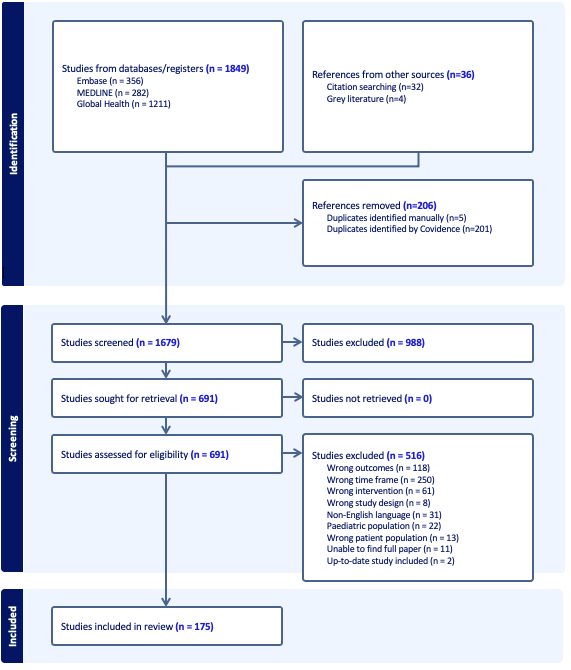


***Appendix 4: PRISMA flow diagram reflecting the process of paper inclusion and exclusion***

*Table 2A.2: Data Extraction Table for Blood Donors*
